# 

**Published:** 1829-04

**Authors:** 


### MONTHLY LIST OF MEDICAL BOOKS.

[*Medical Works cannot be entered on this List except a copy be sent for the purpose*; *the titles of Books having frequently been transmitted to us, as published, which have not appeared for weeks, or even months, after*.]

Illustrations of the Diseases of the Breast. By Sir ASTLEY COOPER, Bart. F.R.S. Serjeant Surgeon to His Majesty; Consulting Surgeon of Guy's Hospital; Lecturer on Anatomy and Surgery, &c. In two Parts. Part I. Coloured Plates.—4to. pp. 89. London, 1829.

A Treatise on Obstructed and Inflamed Hernia, and on Mechanical Obstructions of the Bowels internally; and also an Appendix containing a brief Statement of the Cause of Difference in Size in the Male and Female Bladder. By HENRY STEPHENS, Surgeon.—8vo. pp. 190. London, 1829.

Letters on the Study and Practice of Medicine and Surgery, and on Topics connected with the Medical Profession. Addressed to Students, young Practitioners, and Parents and Guardians. By JAMES WALLACE, Assistant Surgeon, R.N.; Author of "A Voyage to India, &c."—8vo. pp. 210. Glasgow, 1828.

These Letters contain many useful hints for students, and for those who preside over the education of youths destined for the profession.

Medical Botany, No. 27, for March. By JOHN STEPHENSON, M.D. and JAMES MORSS CHURCHILL, F.L.S. &c.

In this Number very elegant plates and correct descriptions are given of the Curcuma Zedoaria, the Fucus vesiculosus, and the Aloe vulgaris.

Annali Universali di Medicina. Fasc. di Gennajo, 1829.—Milano.

A Preliminary Dissertation, illustrative of a new System of Pulmonary Pathology; combining a rational Theory, with a successful Method of conducting the Cure of Consumption. By P. P. P. MYDDELTON, M.D. &c.—Bath, 1825.

### NOTICES.

*The Editors have not received a copy of* Dr. LOUDON's *work.*

*Communications have been received from* Mr. MARSHALL, Mr. BARCOME, *of Demerara, (through* Dr. JAMES JOHNSON,) Mr. JEWEL, R.N., Mr. BURNETT, *and* Mr. GREEN.

*The suggestions of our Correspondent upon the treatment of " Varicose Veins" shall appear in our next Number.*

